# Genomic Selection Outperforms Marker Assisted Selection for Grain Yield and Physiological Traits in a Maize Doubled Haploid Population Across Water Treatments

**DOI:** 10.3389/fpls.2018.00366

**Published:** 2018-03-20

**Authors:** Diego Cerrudo, Shiliang Cao, Yibing Yuan, Carlos Martinez, Edgar Antonio Suarez, Raman Babu, Xuecai Zhang, Samuel Trachsel

**Affiliations:** ^1^Facultad de Agronomia, Universidad Nacional de Mar del Plata, Buenos Aires, Argentina; ^2^Global Maize Program-Physiology, International Maize and Wheat Improvement Center (CIMMYT), Carretera México Veracruz, Texcoco, Mexico; ^3^Maize Research Institute, Heilongjiang Academy of Agricultural Sciences, Harbin, China; ^4^Maize Research Institute, Sichuan Agricultural University, Wenjiang, China

**Keywords:** genomic selection, maize, drought, QTL, DH

## Abstract

To increase genetic gain for tolerance to drought, we aimed to identify environmentally stable QTL in *per se* and testcross combination under well-watered (WW) and drought stressed (DS) conditions and evaluate the possible deployment of QTL using marker assisted and/or genomic selection (QTL/GS-MAS). A total of 169 doubled haploid lines derived from the cross between CML495 and LPSC7F64 and 190 testcrosses (tester CML494) were evaluated in a total of 11 treatment-by-population combinations under WW and DS conditions. In response to DS, grain yield (GY) and plant height (PHT) were reduced while time to anthesis and the anthesis silking interval (ASI) increased for both lines and hybrids. Forty-eight QTL were detected for a total of nine traits. The allele derived from CML495 generally increased trait values for anthesis, ASI, PHT, the normalized difference vegetative index (NDVI) and the green leaf area duration (GLAD; a composite trait of NDVI, PHT and senescence) while it reduced trait values for leaf rolling and senescence. The LOD scores for all detected QTL ranged from 2.0 to 7.2 explaining 4.4 to 19.4% of the observed phenotypic variance with R^2^ ranging from 0 (GY, DS, lines) to 37.3% (PHT, WW, lines). Prediction accuracy of the model used for genomic selection was generally higher than phenotypic variance explained by the sum of QTL for individual traits indicative of the polygenic control of traits evaluated here. We therefore propose to use QTL-MAS in forward breeding to enrich the allelic frequency for a few desired traits with strong additive QTL in early selection cycles while GS-MAS could be used in more mature breeding programs to additionally capture alleles with smaller additive effects.

## Introduction

Agriculture faces the challenge of increasing grain yield of major crops under climate scenarios with higher temperatures and more erratic precipitations as a result of anticipated climate change (Lobell et al., 2011). Climate change will have the strongest detrimental effects on crop production in tropical and subtropical environments since climate change is expected to have larger negative impact than in most other environments (Porter et al., 2014; Rosenzweig et al., 2014). Drought affects approximately 20% of the tropical and subtropical maize produced in developing countries in any given year (Heisey and Edmeades, 1999). Moreover, frequency and intensity of drought are projected to increase in the next decades (Li et al., 2009). Rates of genetic gain are below the ones needed to meet the projected demand in the next few decades in many countries in Sub-Saharan Africa, Central America and Asia (Ray et al., 2013). Under drought, genetic gain is limited by large genotype-by-environment interaction and the complexity of the genetic basis of drought tolerance (Bartels and Sunkar, 2005; Trachsel et al., 2016). Development of maize tolerant to drought that also performs well in non-stressed conditions is essential to ensure food security in the future (Pennisi, 2008).

In the past, secondary traits with strong genetic correlation with grain yield, high heritability, and cost-effective to measure have facilitated the selection in tropical breeding programs (Chapman and Edmeades, 1999; Betrán et al., 2003). Examples include anthesis silking interval (ASI), ears per plant, time to anthesis, leaf rolling, PHT, and senescence (Edmeades et al., 1999; Monneveux et al., 2006). More recently, NDVI measured during canopy development stages, as an indicator for early vigor was proposed as a secondary trait to be included in breeding for maize grain yield under both WW and DS conditions (Trachsel et al., 2016).

Traditional marker-assisted selection using QTL-MAS has been another complementary tool to speed up and make selection more efficient in maize breeding programs (Ribaut and Ragot, 2007; Tuberosa and Salvi, 2009; Beyene et al., 2016). Moreover, several QTL have been identified for grain yield under WW conditions (Messmer et al., 2009, 2011) and drought stressed conditions (Hao et al., 2010; Almeida et al., 2013), for PHT and NDVI (Trachsel et al., 2016), stay green (Almeida et al., 2013) and root traits (Trachsel et al., 2009). However, identification of QTL that are constitutive across environments and populations is essential for use in marker-assisted selection (Bernier et al., 2008). As a result of genotype-by-environment interaction, genetic correlation among traits and QTL detected usually differ among environments (Bolanos and Edmeades, 1996; Tuberosa et al., 2002). Moreover, QTL detected for a trait usually differ among genetic background (Rong et al., 2007) and between inbred line *per se* and their testcross hybrids (Mei et al., 2005; Szalma et al., 2007).

Many QTL studies carried out in the past have limited value for breeding because marker densities and genetic resolution were too low. Recently, however, genotyping-by-sequencing (GBS) has been proposed as an approach to increase the availability of molecular markers from ~100 to thousands of SNP evenly distributed throughout the genome (Elshire et al., 2011; Poland et al., 2012). Thus, the confidence interval surrounding a QTL was reduced, allowing the development of genetic maps with high resolution and precise mapping of QTL.

Marker-assisted selection based on genomic selection (GS-MAS) was highlighted as a new approach for maize breeding (Meuwissen et al., 2001). In GS-MAS, favorable individuals are selected based on genomic estimated breeding values (GEBVs). The major advantage of GS-MAS is that alleles with minor effects can be captured and used in selection (Meuwissen et al., 2001). Both QTL-MAS and GS-MAS require a high marker density in the discovery or training phase, respectively. In the deployment phase QTL-MAS only requires the use of markers flanking the target QTL (more for backcrosses with selection against the genome of the donor outside the area of the target QTL), while GS-MAS requires a higher number of markers adequately covering the entire genome resulting in higher genotyping cost for GS-MAS (Peng et al., 2014).

Simulation and empirical studies indicate that GS-MAS outperforms QTL-MAS for complex traits controlled by many QTL with minor effects or low heritability (Bernardo and Yu, 2007; Mayor and Bernardo, 2009; Heffner et al., 2010; Guo et al., 2013). If adequately integrated in the breeding workflow GS-MAS can partially replace field testing and reduce line development time (Heffner et al., 2010), while QTL-MAS can be used to introgress favorable alleles into an elite background and for integration of (native) traits into a breeding pipeline (Lorenzana and Bernardo, 2009; Zhao et al., 2012; Peng et al., 2014).

A combination of QTL-MAS and GS-MAS has also been suggested as an integration of knowledge on functional markers as either known causative mutations or known QTL associations with yet to be identified genes, for improved prediction (Zhao et al., 2012; Jonas and De Koning, 2016; Cao et al., 2017). These propositions suggest the importance of flexible GS as a method for introduction into breeding programs and combining it with QTL-MAS (Nakaya and Isobe, 2012). For both QTL-MAS and GS-MAS the success depends on excellent phenotypic characterization during the discovery or training phase, respectively.

In an aim to better understand the genetic architecture of drought tolerance and to evaluate the suitability of QTL-or GS-MAS for selection toward drought tolerance, we used genotyping-by-sequencing technology to detect QTL and to develop GS models for grain yield and secondary traits in a DH population in *per se* and testcross evaluations, under WW and DS conditions. The specific objectives of this study were to: (i) evaluate QTL consistency across *per se* and testcross evaluations under WW and DS conditions, and detect QTL that are constitutive across studies with related populations; (ii) compare how QTL mapping and GS are affected by population (*line per se* vs. testcrosses) and treatments (WW vs. DS) and strategize their deployment in a drought breeding program.

## Materials and methods

### Plant material

A bi-parental DH line population, consisting of 169 genotypes and the testcross hybrids, consisting of 190 genotypes was evaluated. The DH population was derived from an F1 cross between drought tolerant lines, La Posta Sequia C7-F64-2-6-2-1-B-B (LPSC7F64), and an elite inbred line from CIMMYT, CML495 (Cairns et al., 2013). The first parental line is traced to the La Posta Sequia Population (LPS), a white dent, Tuxpeño-related synthetic, well adapted to lowland tropics. Full sib recurrent selection was carried out under drought conditions from cycle 0 to cycle 7. The second parental line is a white flint line described as late-lowland with tolerance to rust, helminthosporium, good standability, belonging to heterotic group A. The lines were testcrossed to CML494 for the phenotypic evaluation.

### Experimental design and environmental conditions

Each of the populations (i.e., hybrids and lines) was evaluated under well-watered (WW) and drought stressed (DS) conditions in experiments conducted in different locations in Mexico during winter cycles of 2013, 2014, and 2016 (Table 1). A total of 11 experiments were conducted in Iguala (Guerrero, Mexico; IG), Agua Fria (Puebla, Mexico; AF) and Tlatizapan (Morelos, Mexico; TL); three for hybrids-WW (IG2013, AF2016, TL2016), four for hybrids-DS (IG2013, TL2013, TL2014, TL2016), two for lines-WW (IG2013, TL2013) and two for lines-DS (IG2013, TL2013).

**Table 1:** Summary of experiments describing their location, season, population, treatment (well-watered, WW; and drought stressed conditions, DS), planting date (PD), seasonal precipitation, mean seasonal temperatures (T), fertilization rates and latitude, longitude and altitude above sea level (asl).

**Location**	**Season**	**Population**	**Treatment**	**PD**	**Precipitation**	**T**	**Fertilizaction**	**Latitude**	**Longitude**	**asl**
					**(mm)**	**(°C)**	**(NPK, kg/ha)**	**(°N)**	**(°W)**	**(m)**
Iguala	2013	Hybrids	WW	29/11/2012	115	23.6	160/60/25	18.349	99.508	732
Agua Fria	2016	Hybrids	WW	6/12/2015	55	22.6	150/80/30	27.455	97.640	110
Tlaltizapan	2016	Hybrids	WW	24/12/2015	41	21.8	160/60/25	18.679	99.130	945
Iguala	2013	Hybrids	DS	29/11/2012	115	23.6	160/60/25	18.349	99.508	732
Tlaltizapan	2013	Hybrids	DS	11/12/2012	267	24.1	160/60/25	18.679	99.130	945
Tlaltizapan	2014	Hybrids	DS	16/12/1013	52	20.8	160/60/25	18.679	99.130	945
Tlaltizapan	2016	Hybrids	DS	19/12/2015	41	21.6	160/60/25	18.679	99.130	945
Iguala	2013	Lines	WW	29/11/2012	115	23.6	160/60/25	18.349	99.508	732
Tlaltizapan	2013	Lines	WW	11/12/1012	267	24.1	160/60/25	18.679	99.130	945
Iguala	2013	Lines	DS	29/11/2012	115	23.6	160/60/25	18.349	99.508	732
Tlaltizapan	2013	Lines	DS	11/12/1012	267	24.1	160/60/25	18.679	99.130	945

For all the experiments, the design was an alpha-lattice (0, 1) replicated twice with incomplete blocks size of 5. Plots consisted of one row 4.5 m long at row spacing of 0.75 m. Plots were hand-seeded with two seeds per hill and thinned to one plant per hill (22 plants per plot; 6.6 plants/m^2^) three weeks after planting.

For the DS treatment, water deficit was induced by withholding irrigation 12-15 days (~190 GDD) before flowering with the aim of reaching the permanent wilting point at flowering at 40 cm soil depth. In the case of severe drought, as indicated by an ASI above 5 d on trial average, irrigation was applied 7 d after completion of anthesis, while a second irrigation was applied 3 wk after completion of anthesis. In the case of moderate DS, only one irrigation was applied during the grain filling period, 2 wk after completion of anthesis. For the WW experiments, evapotranspirated water was fully compensated for through weekly irrigations. Soil moisture content was measured at 10, 20, 30, 40, 60, and 100 cm soil depth three times weekly using Delta-t PR2/6 soil moisture probes (Delta-T devices, Cambridge, United Kingdom) to schedule irrigations in the drought stress treatment. Fertilization, insecticides and herbicides were applied as needed. Fertilizer quantities applied at each location are reported in Table 1.

### Phenotypic data acquisition

Several phenotypic traits were measured in each plot throughout the growing season. Two, four, five, and six weeks after planting the NDVI was measured using an RT-505 Greenseeker (Trimble, Ukia, CA, USA). NDVI measurements were taken by running the sensor in the middle of each plot at a height of 80 cm above the canopy. NDVI was calculated per the following equation: R_NIR_-R_Red_/R_NIR_+R_Red_, where R_NIR_ is the reflectance of near infrared wavelength, and R_Red_ is the reflectance of red wavelength. The NDVI illustrates the part of red wavelength which is absorbed by the plant. At flowering, anthesis and silking dates were recorded when 50% of plants within a plot were shedding pollen and growing silks, respectively. The ASI was calculated as the difference between female and male flowering dates. Two, four and six weeks after flowering senescence was measured visually using a scale ranging from 1 (no senescence) to 9 (complete senescence) to approximate stay green (Trachsel et al., 2016). Leaf rolling was measured visually at flowering, and one and two weeks after flowering using a scale ranging from 1 (unrolled, turgid) to 5 (rolled, onion leaf). For NDVI, leaf rolling and senescence, the area under the curve was calculated by integrating a polynomial function of second degree fitted to individual measurements taken before (for NDVI) or after flowering (for senescence and leaf rolling). In this study, a new secondary trait indicative of early vigor, senescence and overall green leaf area and duration (GLAD) is proposed and evaluated. GLAD was calculated as:

$$\begin{array}{l} \text{GLAD}=\left(\text{PHT x NDVI}\right)/\text{senescence} \end{array}$$

were PHT is plant height at flowering and NDVI and senescence are area under the curve of four NDVI readings and three senescence scores as described above. The area under the curve (AUC) for NDVI and senescence was calculated by integrating a polynomial function of second degree fitted to individual measurements taken before (for NDVI) or after flowering (for senescence).

After physiological maturity was reached, all ears of each plot were collected and shelled, grain moisture was recorded. Grain yield is reported at 12% moisture.

### Phenotypic data analysis

The mixed effect linear model used for the analysis of phenotypic data measured in multilocation trials was:

$$\begin{array}{l} {\text{Y}}_{\text{hmlk}}=\mu +\alpha_{\text{h}}+{\text{E}}_{\text{ml}}+\alpha_{\text{hEml}}+{\text{r}}_{\text{m}}\left({\text{E}}_{\text{ml}}\right)+{\text{r}}_{\text{m}}\left({\text{E}}_{\text{ml}}\right)\delta_{\text{k}}+{\text{e}}_{\text{hmlk}} \end{array}$$

Where Y_hmlk_ is the trait value of the h_th_ genotype (h = 190 and 169 for hybrids and lines, respectively) for the l_th_ location (hybrids-WW: l = 3; hybrids-DS: l = 4, lines-WW: I = 2; lines-DS: I = 2), the m_th_ replication (*m* = 2); μ the overall mean, α_h_ the main effect of the genotype, E_ml_ the effect of the location, α_hEml_ the genotype-by-location interaction, r_m_(E_ml_) the replication within location effect and r_m_(E_ml_)δ_k_ the effect of blocks within replicates within locations and the random error term e_hmlk_. All factors except μ were set as random. Best linear unbiased predictors (BLUPs) of genotypes, variance components, and broad sense heritability were obtained. Data for each population-by-treatment combination were analyzed separately. Analysis of the genotype-by-treatment (i.e., WW and DS) interactions was carried out separately for hybrids and lines. For that, terms for treatment and genotype-by-treatment interaction were added to the model described before. Location, genotype and treatment were set as fixed and the rest as random factors. Plant stand was used as a covariate for grain yield and NDVI calculations. Plots with less than 18 plants were removed from the analysis. Variance components were estimated by restricted maximum likelihood (REML) and heritability as the relationship between genetic and phenotypic variance, according to the formula:

$$\begin{array}{l} h^{2}=(\sigma_{\text{G}}^{2})/((\sigma_{\text{G}}^{2}+({\sigma_{\text{GxE}}}^{2}/l)+e/(r^{*}l)) \end{array}$$

where $\sigma_{\text{G}}^{2}$ is the genotypic variance, ${\sigma_{\text{GxE}}}^{2}$ the genotype-by-environment interaction variance, e the error term, l the number of environments and r the number of replications within environments.

BLUPs for genotypes effects are shrinkage predictors obtained as:

α~=GˆZ'Vˆ-1(y-1μ)

using matrix notation, where y is the vector of the response variable, G ˆ the matrix of variance covariance of the random effects, Z the design matrix for random effects in the model, V ˆ estimated variance of y, 1 a vector of ones and μ the overall mean, the only fixed parameter in the model. The cor.test function in R was used to calculate correlations among BLUPs. Genetic correlations among traits were estimated with a method described previously (Cooper and Delacy, 1994).

### Genotyping and linkage map construction

For all the maize lines tested in this study, leaf samples bulked from 12 plants of each line were used for DNA extraction with a Cetyltrimethylammonimum bromide (CTAB) procedure (CIMMYT, 2005). A genotyping-by-sequencing (GBS) protocol commonly used by the maize research community was applied at the Cornell University Biotechnology Resource Center in this study (Elshire et al., 2011; Wu et al., 2016). Briefly, the GBS libraries were constructed in 96-plex, and genomic DNA was digested with the restriction enzyme ApeK1. Each library was sequenced on a single lane of Illumina flow cell. SNP calling was performed using TASSEL 5.0 GBS Discovery Pipeline with B73 as the reference genome. Initially, 955,690 SNPs evenly distributed on maize chromosomes were called for each line; 955,120 of them were assigned to chromosomes 1–10, and 570 of them could not be anchored to any of the 10 maize chromosomes. A bin map was constructed by using 20,473 high quality filtered GBS SNPs, details on how to construct the linkage map were described previously (Cao et al., 2017). In brief, neighbor SNPs having high similarity haplotype information were clustered into one bin, and each bin was treated as single marker to construct the genetic map. The following steps were performed to reduce genotyping error and eliminate the low quality SNPs from the bin map: (1) DH lines with heterozygosity rate greater than 5% and/or missing rate greater than 20% were eliminated from further analysis; (2) unlinked SNPs were removed from further analysis, where the window size was 8, similarity rates of all the SNPs within each window were calculated to remove the unlinked SNPs, threshold of similarity rate was 95%; (3) the consecutive SNPs with high similarity rate, i.e., 95%, were merged into one bin; and (4) bins were treated as genetic markers to construct a genetic map. The genetic map was constructed with 191 bins in software QTL IciMapping Version 4.0 (www.isbreeding.net; Wang et al., 2014). The total genetic map length was 987.35 cM resulting in an average distance between markers of 5.15 cM.

### Genomic selection analysis

Genomic prediction was implemented in rrBLUP package (Endelman, 2011) in DH population. SNPs in the genetic map were used for genomic prediction. Details of the implementation of rrBLUP were described earlier (Zhao et al., 2012). A five-fold cross-validation scheme with 100 replications was used to generate the training and validation sets and assess the prediction accuracy. The average value of the correlations between the phenotype and the genomic estimated breeding values was defined as genomic prediction accuracy (r_MG_).

## Results

### Heritability, phenotypic data and correlations between grain yield and secondary traits

A population of DH lines was evaluated *per se* and in testcross combination under WW and DS conditions. Significant genotype-by-location interaction was detected when experiments were combined by population and irrigation treatment (i.e., hybrids-WW and hybrids-DS; lines-WW and lines-DS) for all traits, with exception of PHT for lines under DS (Table 2). For most traits, heritability of the combined analysis remained acceptably high. Heritability of PHT and anthesis were the highest in most experiments, with values above 0.75. The ASI had the lowest values, ranging from 0.17 to 0.76. Under DS conditions, grain yield, NDVI and GLAD had the highest heritability values, ranging from 0.45 to 0.81. Meanwhile, senescence and leaf rolling had the lowest heritability, ranging from 0.14 to 0.66.

**Table 2:** Mean, 1st and 3rd quartile, heritability, genotype effect and genotype-by-location interaction (g^*^l), for different traits evaluated in experiments that included hybrids under well-watered (Hybrids_WW), hybrids under drought stress (Hybrids_DS), lines under well-watered (Lines_WW) and lines under drought stress conditions (Lines_DS).

** Experiment**		**Trait**								
		**GY (t/ha)**	**AD (days)**	**PHT (cm)**	**ASI (days)**	**NDVI**	**SEN**	**LR**	**GLAD**	**DSS (%)**
Hybrids_WW	Mean	7.02	81.8	221	−0.11	15.9				63.0
	SE	0.65	1.43	7.2	1.26	0.99				*NA*
	1st quartile	6.71	81.2	218	−0.29	15.7				61.3
	3rd quartile	7.39	82.4	225	0.04	16.1				64.8
	h^2^	0.70	0.78	0.75	0.52	0.51				
	genotype	^***^	^***^	^***^	^***^	^***^				
	g^*^l	^***^	^***^	^***^	^***^	^***^				
Hybrids_DS	Mean	2.59	83.1	195	1.83	14.1	127	52.9	22.6	
	SE	0.38	0.87	0.35	0.86	0.71	6.9	4.96	1.67	
	1st quartile	2.51	82.7	194	1.78	13.9	126	52.5	22.3	
	3rd quartile	2.68	83.5	196	1.88	14.3	128	53.1	23.0	
	h^2^	0.37	0.65	0.31	0.17	0.57	0.38	0.14	0.53	
	genotype	^**^	^**^	^**^	^*^	^**^	^***^	ns	^***^	
	g^*^l	^***^	^***^	^***^	^***^	^***^	^***^	^**^	^***^	
Lines_WW	Mean	2.07	87.4	112	0.61	11.6				63.8
	SE	0.28	*NA*	8.2	2.59	0.69				*NA*
	1st quartile	1.89	86.3	107	0.38	11.4				60.2
	3rd quartile	2.25	88.5	117	0.82	11.7				68.7
	h^2^	0.52	0.79	0.81	0.30	0.45				
	genotype	^***^	^***^	^***^	^*^	^***^				
	g^*^l	^***^	^***^	^***^	^***^	^***^				
Lines_DS	Mean	0.75	89.6	102	1.07	10.9	142	69.0	8.14	
	SE	0.21	1.5	7.49	1.42	0.18	11.2	5.56	0.26	
	1st quartile	0.63	88.0	98.5	1.81	10.6	139	66.3	7.46	
	3rd quartile	0.85	91.3	106	0.20	11.3	145	71.4	8.65	
	h^2^	0.76	0.91	0.80	0.76	0.71	0.61	0.66	0.81	
	genotype	^***^	^***^	^***^	^***^	^***^	^***^	^***^	^***^	
	g^*^l	^***^	^***^	ns	^*^	^**^	^***^	^***^	^***^	

*Traits measured included grain yield (GY), anthesis date (AD), plant height (PHT), anthesis-silking interval (ASI), normalized differential vegetative index (NDVI), senescence (SEN), leaf rolling (LR), GLAD and drought stress susceptibility (DSS)*.

ns, *, **, *** *non-significant, and significant at p < 0.1, 0.05, and 0.01, respectively*.

Grain yield for lines and hybrids was on average reduced by 63% under DS relative to WW (Table 2). Drought stress equally increased ASI (1.94 and 0.38 days for hybrids and lines, respectively), decreased PHT (26 and 10 cm for hybrids and lines, respectively) and delayed anthesis (1.8 and 2.2 days for hybrids and lines, respectively). Differences in NDVI between WW and DS before the onset of drought, can be explained by differences in environments as a result of unbalanced experimental data. Drought trials were all carried out in the winterseason (with lower temperatures compared to the summerseason), while the non-stressed trials were grown in both the summer and winterseason. Since the crop typically develops slower in winter and NDVI readings were taken in calendar days after planting, plants were on average less developed in Winter, relative to plants grown in trials carried out in both summer and winter, resulting in lower NDVI values.

DS were measured at completely dry locations (~900–1,100 masl) whereas additional WW treatments were included in locations with higher precipitations.

Senescence, leaf rolling and GLAD were only recorded under DS conditions. Averaged across treatments, hybrids reached anthesis six days earlier, grew 101 cm taller and had 24% higher NDVI than lines. Leaf rolling, and GLAD were 30 and 177% higher in the hybrids than in the lines and senescence was 11% higher for the lines. The ASI did not differ among lines and hybrids (*p* > 0.05).

Correlations among grain yield and secondary traits differed across populations and irrigation treatments (Table 3). Grain yield was moderately correlated to NDVI (*r*_g_ = 0.84; *r*_p_ = 0.53) and GLAD (*r*_g_ = 0.70; *r*_p_ = 0.49), for hybrids-WW and hybrids-DS, respectively. Grain yield correlated moderately to weakly with PHT, with highest correlation coefficients (*r*_g_ = 0.67; *r*_p_ = 0.54) for hybrids-WW. Correlations with anthesis, ASI, leaf rolling and senescence were weak or even non-significant for some trait-by-treatment combinations.

**Table 3:** Phenotypic (r_p_) and genotypic (r_g_) correlations between grain yield and different secondary traits for lines *per se* and their testcross hybrids under well-watered (WW) and drought stressed (DS) conditions.

**Trait**	**Hybrids_WW**		**Lines_WW**	
	**r_p_**	**r_g_**	**r_p_**	**r_g_**
Anthesis	ns	ns	ns	ns
PHT	0.54^***^	0.67^***^	0.26^***^	0.25^***^
ASI	ns	ns	−0.22^***^	−0.66^***^
NDVI	0.53^***^	0.84^***^	0.53^***^	0.70^***^
	**Hybrids_DS**		**Lines_DS**	
Anthesis	−0.20^***^	ns	−0.18^***^	−0.21^***^
PHT	0.29^***^	ns	0.36^***^	0.38^***^
ASI	−0.27^***^	ns	−0.14^***^	−0.21^***^
NDVI	0.42^***^	0.72^***^	0.48^***^	0.66^***^
Senecence	−0.11^*^	ns	−0.32^***^	−0.25^***^
Leaf rolling	−0.20^***^	ns	−0.42^***^	−0.52^***^
GLAD	0.49^***^	0.70^***^	0.60^***^	0.61^***^

*Traits evaluated included anthesis, plant height (PHT), anthesis-silking interval (ASI), normalized differential vegetative index NDVI, senescence, leaf rolling and GLAD*.

ns,*, **, *** *Correlations non-significant, significant at p < 0.1, 0.05, and 0.01, respectively*.

### Detected QTL for grain yield and secondary traits; collocation in bins 1.02, 1.03, and 7.04

The analysis revealed a total of 48 significant QTL for nine traits evaluated (Table 4). They included 13 QTL detected in hybrids-WW, 12 in hybrids-DS, 12 in lines-WW and nine in lines-DS. Thirteen QTL were detected for PHT, eight for grain yield, seven for anthesis, six for senescence, four for ASI, three for GLAD and two each for leaf rolling, NDVI and DSS. In most cases, the allele derived from CML495 increased trait values for anthesis, ASI, PHT, NDVI, DSS, and GLAD, while it reduced trait values for DSS, leaf rolling and senescence. The LOD scores for all detected QTL ranged from 2.0 (grain yield, hybrids-DS) to 7.2 (grain yield, lines-WW) explaining 4.4 (grain yield, lines-WW) to 19.4% (grain yield, lines-WW) of the observed phenotypic variance. Only one constitutive QTL for grain yield was detected, which mapped to bin 8.08 for lines in WW and DS. The trait increasing allele was derived from LPSC7F64 in both cases. None of the 39 QTL detected for secondary traits overlapped for hybrid and line or across treatments.

**Table 4:** Summary of all QTL detected in experiments (Exp) of hybrids (HY) and lines (LI), under well-watered (WW) and drought stressed (DS) conditions showing chromosome (Chr), position (Pos), bin, flanking markers, LOD scores, phenotypic variance explained by a QTL (PVE), and additive effects.

**Trait**	**Exp**	**Chr**	**Pos (cM)**	**Bin**	**Left marker**	**Right marker**	**LOD**	**PVE (%)**	**Add**
DSS	Hybrids	1	44	1.02	1_26208604	1_28662442	2.48	7.42	−1.12
Anthesis	HYDS	1	40	1.02	1_22101580	1_26208604	3.91	10.95	−0.26
GLAD	HYDS	1	33	1.02	1_14260188	1_18734111	2.10	4.73	−0.16
PHT	HYWW	1	43	1.02	1_22101580	1_26208604	2.48	6.85	−1.55
NDVI	HY	1	70	1.03	1_49826154	1_54856976	4.62	13.06	−0.13
GLAD	HYDS	1	64	1.03	1_42290528	1_46373739	2.11	4.81	−0.16
Grain yield	HYWW	1	61	1.03	1_42290528	1_46373739	3.13	8.97	−0.18
PHT	HYWW	1	64	1.03	1_42290528	1_46373739	4.32	9.87	−2.89
Anthesis	LIDS	1	58	1.03	1_37544296	1_42290528	3.72	13.85	−1.07
ASI	LIWW	1	73	1.04	1_54856976	1_59889149	2.52	8.66	−0.13
PHT	LIWW	1	73	1.04	1_54856976	1_59889149	2.68	5.80	−2.10
Anthesis	HYWW	1	109	1.06	1_193139090	1_197536500	3.71	11.01	0.30
Grain yield	HYDS	1	139	1.07	1_216309112	1_222372321	2.00	6.34	−0.03
PHT	HYWW	1	117	1.07	1_200801163	1_200801163	2.27	5.78	1.43
NDVI	LI	1	137	1.07	1_216309112	1_222372321	2.06	5.40	−11.3
ASI	HYWW	2	21	2.02	2_6452607	2_7335967	2.03	4.83	−0.08
PHT	LIWW	2	101	2.07	2_194130021	2_195513479	5.48	11.84	3.06
PHT	HYWW	2	132	2.08	2_218568786	2_222185087	2.93	6.51	2.35
Senecence	HYDS	2	164	2.09	2_233065497	2_236696694	3.90	11.56	0.57
PHT	HYWW	2	164	2.09	2_233065497	2_236696694	2.57	5.86	2.00
Senecence	HYDS	3	15	3.01	3_3103988	3_3809626	2.25	6.67	0.44
DSS	Lines	3	53	3.04	3_27522260	3_39144097	2.17	8.02	−1.73
PHT	HYWW	3	61	3.05	3_123719230	3_139771507	2.03	5.16	−1.35
Senecence	LIDS	3	105	3.06	3_179531424	3_182811545	3.80	14.80	2.03
ASI	HYWW	4	48	4.03	4_16118475	4_19556036	2.44	6.72	0.09
Grain yield	LIWW	4	54	4.04	4_24377671	4_61369128	3.16	7.25	0.08
Senecence	LIDS	4	56	4.05	4_61369128	4_116190231	3.10	12.01	−1.84
PHT	LIWW	4	60	4.06	4_148669865	4_155298867	5.43	11.64	2.96
ASI	HYDS	4	116	4.08	4_185251502	4_186037532	4.57	12.35	−0.03
Grain yield	LIWW	4	106	4.08	4_180189654	4_181422373	2.05	4.45	0.06
Anthesis	LIDS	4	137	4.09	4_232389072	4_233931750	2.30	7.42	−0.79
Anthesis	LIWW	4	148	4.10	4_237578508	4_238180236	2.90	9.41	−0.62
Grain yield	LIWW	4	158	4.10	4_238612431	4_239603458	7.25	19.41	0.13
Anthesis	LIWW	5	100	5.07	5_206242623	5_207497380	2.19	6.68	0.51
Senecence	LIDS	5	48	5.09	5_77670149	5_97282620	2.79	10.74	−1.78
PHT	LIDS	6	15	6.08	6_60180411	6_89140482	2.32	8.73	−1.87
Grain yield	HYWW	7	61	7.02	7_89338077	7_109910472	2.03	5.36	−0.14
GLAD	HYDS	7	74	7.03	7_128254490	7_129764113	2.62	6.39	−0.19
PHT	LIWW	7	87	7.03	7_134468855	7_136543672	5.32	11.40	−2.93
Leaf rolling	HYDS	7	126	7.04	7_165776873	7_166705322	3.19	9.21	0.14
Senecence	HYDS	7	117	7.04	7_162474709	7_164294427	2.99	8.77	0.51
PHT	HYWW	7	112	7.04	7_159247704	7_161345394	4.10	11.58	−2.06
PHT	HYDS	8	95	8.06	8_155841571	8_162287143	2.43	7.23	−0.33
Grain yield	LIDS	8	138	8.08	8_171722422	8_172044417	2.05	7.28	0.06
Grain yield	LIWW	8	137	8.08	8_171722422	8_172044417	2.50	5.42	0.07
Grain yield	HYDS	9	0	9.00	9_1265981	9_2794057	2.33	7.29	0.03
Anthesis	LIDS	9	110	9.07	9_151402029	9_152104326	2.22	7.11	−0.77
Leaf rolling	LIDS	10	65	10.05	10_134751974	10_135546981	2.45	8.67	1.43

*Traits include normalized differential vegetative index NDVI, anthesis, anthesis-silking interval (ASI), plant height (PHT), senescence, GLAD, grain yield, and drought stress susceptibility (DSS)*.

A collocation of QTL for grain yield (hybrids-WW), anthesis (lines-DS), PHT (hybrids-WW), NDVI (hybrids-WW), and GLAD (hybrids-DS) was detected in bin 1.03 (Table 4). The phenotypic variance explained by each QTL ranged from 4.8 (GLAD, hybrids-DS) to 13.8% (NDVI, hybrids-WW). The allele derived from CML495 delayed anthesis by 1.07 d, increased GLAD by 0.16, grain yield by 0.18 t/ha, NDVI by 0.13 and PHT by 2.89 cm. Another collocation was identified in bin 1.02, where QTL for DSS (hybrids), anthesis (hybrids-DS), PHT (hybrids-WW) and GLAD (hybrids-DS) were detected. The phenotypic variance explained by those QTL ranged from 4.7 (GLAD, hybrids-DS) to 10.9% (anthesis, hybrids-DS). The allele derived from CML495 delayed flowering by 0.26 d, increased GLAD by 0.16, PHT by 1.55 cm and DSS by 1.12%. Another collocation of QTL for PHT (hybrids-WW), leaf rolling (hybrids-DS) and senescence (hybrids-DS) detected in bin 7.04 is indicative of a beneficial effect of early vigor when stress occurs during the post flowering period. The allele derived from CML495 increased PHT and decreased leaf rolling and senescence.

The positive effect of a short ASI on grain yield was confirmed by a collocation of repulsive QTL for ASI and grain yield in bin 4.08 (Table 4). A collocation of repulsive QTL for anthesis and grain yield in bin 4.10 is indicative of the contribution of this chromosomal region to drought escape by early flowering. The grain yield QTL in this bin had the largest phenotypic variance explained among all detected QTL in this study (19.4%), with the trait increasing allele derived from LPSC7F64.

### R^2^of QTL and prediction accuracy of GS models for grain yield and secondary traits

**Figure 1:**
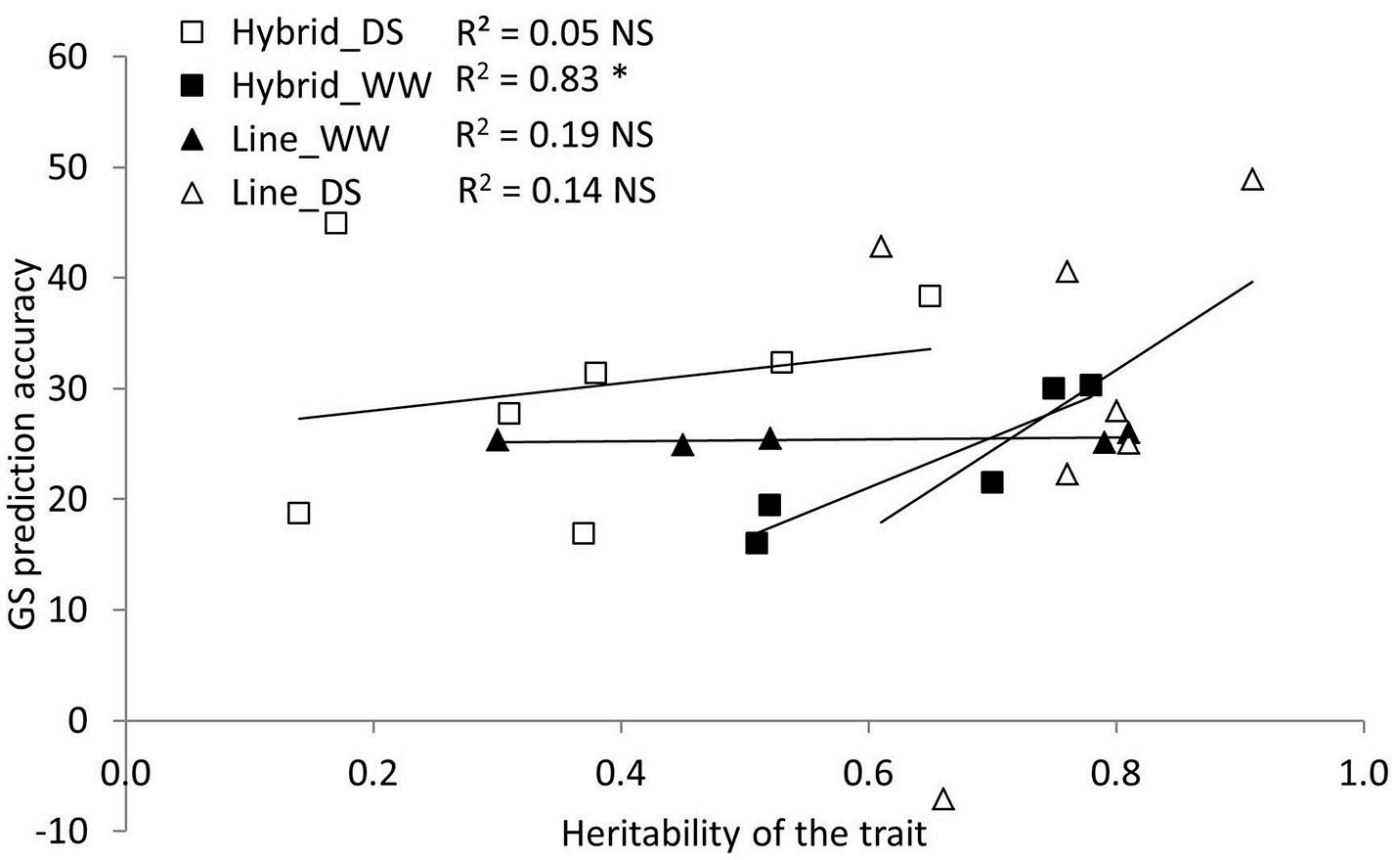
Genomic selection prediction accuracy as a function of the heritability for different traits of maize hybrids under well-watered (Hybrids_WW), hybrids under drought stress (Hybrids_DS), lines under well-watered (Lines_WW) and lines under drought stress conditions (Lines_DS). Traits measured included grain yield, anthesis date, plant height, anthesis-silking interval, normalized differential vegetative index, senescence, leaf rolling, GLAD and drought stress susceptibility.

The variation (R^2^) explained by all QTL for a single trait-by-experiment combination was moderate for grain yield (25.1%, lines-WW), PHT (37.3%, lines-WW) and senescence (25.2%, lines-DS), indicating that genetic control was well captured and is potentially usable in QTL-MAS (Table 5). Lower *R*^2^ values for the rest of the trait-by-experiment combinations indicate that traits are controlled by many minor effect QTL and genotype-by-environment interactions are high, which are not suitable for QTL-MAS. The prediction accuracy of GS models was larger than the *R*^2^ values for grain yield and secondary traits, for hybrids and lines both under WW and DS conditions; except for grain yield and ASI in lines-WW and for leaf rolling in lines-DS. The advantages of the GS-MAS over the QTL-MAS approach were larger under DS than under WW conditions for grain yield, anthesis and PHT as indicated in differences between *R*^2^ (QTL-MAS) and prediction accuracies (GS-MAS); for instance, prediction accuracy of GS and *R*^2^ values for grain yield were 16.9 vs. 0 and 22.3 vs. 0 for hybrids-DS and lines-DS, respectively. Moreover, the prediction accuracy of grain yield under WW was better than under DS (23.5 and 19.6 for the average of hybrids and lines under WW and DS, respectively) and the prediction of most of the secondary traits were better than for GY except for lines-WW. A similar trend was also observed for the secondary traits. A positive correlation was observed between the genomic prediction accuracy and trait heritability for hybrids-WW (*R*^2^ = 0.83; *p* < 0.02, Figure 1). For the other treatment-by-population combinations the correlation between genomic prediction accuracy and trait heritability was low.

**Table 5:** Total phenotypic variance explained by all QTL detected for individual traits (R^2^) and prediction accuracy of genomic selection models measured in hybrids and lines *per se* under well-watered (WW) and drought stressed (DS) conditions.

	**Phenotypic var. explained by all QTL; R**^**2**^				**GS prediction accuracy**			
**Trait**	**Hybrid_WW**	**Hybrid_DS**	**Line_WW**	**Line_DS**	**Hybrid_WW**	**Hybrid_DS**	**Line_WW**	**Line_DS**
GY	8.59	0	25.1	0	21.5	16.9	25.5	22.3
DSS	6.78	NA	0	NA	20.6	NA	0.25	NA
Anthesis	10.4	9.57	9.1	11.8	30.3	38.3	25.2	48.9
PHT	17	6.45	37.3	0	30.0	27.7	26.0	28.0
ASI	0	12.4	7.7	0	19.5	44.9	25.3	40.6
NDVI	12.2	NA	0	NA	16.0	NA	25.0	NA
SEN	NA	14.4	NA	25.2	NA	31.4	NA	42.9
LR	NA	8.59	NA	7.84	NA	18.7	NA	−7.1
GLAD	NA	7.07	NA	0	NA	32.3	NA	25.1

*Traits displayed are: grain yield (GY), drought stress susceptibility (DSS), anthesis, plant height (PHT), anthesis silking interval (ASI), normalized differential vegetative index NDVI, senescence (SEN), leaf rolling (LR) and GLAD*.

### Hybrids with best yield potential and yield stability

The best ten hybrids for WW and DS conditions out-yielded the commercial check (DK357) and the trial mean by 12 and 13% under WW, and by 18 and 11% under DS conditions, respectively (Table 6). Although the genotype-by-water treatment interaction was significant (data not shown) three hybrids with outstanding yield potential and stability (i.e., good performance under WW and DS) were identified (Table 6). The hybrids (LPSC7F64/CML495)DH220/CML494, (LPSC7F64/CML495)DH290/CML494 and (LPSC7F64/ CML495)DH30/CML494, ranked 1st, 2nd, and 3rd under WW conditions, and 22nd, 18^th^, and 13th, out of 190 under DS conditions, respectively. On average, the three hybrids with high yield potential and yield stability across environments out yielded the commercial check and the trial mean by 12 and 13% under WW and by 12 and 6% under DS, respectively.

**Table 6:** Grain yield (t/ha) for the top 10 performing hybrids, the local check (DK-357) and the mean of all evaluated hybrids, under well-watered (WW) and drought stressed (DS) conditions, and grain yield for three hybrids with best combination of potential and grain yield stability, also under well-watered and drought stressed conditions.

**Top 10 under WW**			**Top 10 under DS**			**Best for potential—stability combination**		
**Hybrid ID**	**WW**	**DS**	**Hybrid ID**	**DS**	**WW**	**Hybrid ID**	**WW**	**DS**
(LPSC7F64/CML495)DH220/CML494	8.43	2.75	(LPSC7F64/CML495)DH243/CML494	2.96	7.4	(LPSC7F64/CML495)DH220/CML494	8.43	2.75
(LPSC7F64/CML495)DH265/CML494	8.20	2.51	(LPSC7F64/CML495)DH56/CML494	2.93	6.9	(LPSC7F64/CML495)DH290/CML494	7.99	2.76
(LPSC7F64/CML495)DH290/CML494	7.99	2.76	(LPSC7F64/CML495)DH40/CML494	2.93	7.3	(LPSC7F64/CML495)DH30/CML494	7.85	2.79
(LPSC7F64/CML495)DH13/CML494	7.91	2.73	(LPSC7F64/CML495)DH95/CML494	2.91	7.4			
(LPSC7F64/CML495)DH87/CML494	7.88	2.60	(LPSC7F64/CML495)DH24/CML494	2.87	7.5			
(LPSC7F64/CML495)DH15/CML494	7.85	2.62	(LPSC7F64/CML495)DH257/CML494	2.86	7.4			
(LPSC7F64/CML495)DH241/CML494	7.85	2.73	(LPSC7F64/CML495)DH119/CML494	2.82	7.1			
(LPSC7F64/CML495)DH30/CML494	7.85	2.79	(LPSC7F64/CML495)DH30/CML494	2.82	7.4			
(LPSC7F64/CML495)DH11/CML494	7.84	2.68	(LPSC7F64/CML495)DH127/CML494	2.82	7.0			
(LPSC7F64/CML495)DH9/CML494	7.83	2.56	(LPSC7F64/CML495)DH282/CML494	2.80	7.5			
Top 10 mean	7.96	2.67		2.87	7.28			
Commercial check	7.12			2.43				
All trial mean	7.04			2.59				

## Discussion

We created contrasting WW and DS conditions for the *per se* evaluation of DH lines and in testcross combination. The grain yield reduction of 63% under DS compared to WW conditions was similar to the ones reported for experiments following the same protocols, with a related hybrid population (Trachsel et al., 2016). Moderate to severe drought stress levels allowed us to detect QTL across experiments and treatments (i.e., hybrids-WW, hybrids-DS, lines-WW and lines-DS) and to identify lines and hybrids with good performance across treatments.

Both PHT and NDVI were positively correlated with grain yield under WW and DS conditions, supporting their potential utility for indirect selection for improved grain yield under drought stress as suggested previously (Messmer et al., 2011; Trachsel et al., 2016). GLAD had large positive correlations with grain yield both for lines and hybrids (only measured under DS conditions). GLAD integrates information on different morpho-physiological traits related to grain yield (i.e., early and general vigor and senescence) and could be used to identify genotypes that better combine those traits. Since neither PHT nor senescence were correlated with grain yield for hybrids, it is likely that the positive correlation was caused by large NDVI.

### Beneficial effects of early vigor and escape on grain yield evidenced in bins 1.02, 1.03, and 7.04

A collocation of QTL for anthesis (hybrids-DS), PHT (hybrids-WW), GLAD (hybrids-DS), and DSS (hybrids) is indicative of the importance of bin 1.02 for the genetic control of grain yield and early vigor. Detection of QTL for anthesis and for DSS in this region indicates that the physiological mechanism conveying grain yield under drought stressed conditions is drought escape achieved through an earlier flowering. The importance of this bin is further supported by collocations with QTL detected for anthesis (Salvi et al., 2011) and PHT (Schön et al., 1993) in other genetic backgrounds. Collocations of QTL were detected for PHT, NDVI and senescence, which are all components of GLAD, in the same bin (Trachsel et al., 2016). Two candidate genes potentially accounting for the detected QTL are *ct2* (compact plant2) and *cfr1* (coupling factor reduction1). The first gene is involved in CLAVATA signaling, which controls shoot meristem size and shoot growth (Bommert et al., 2013), while *cfr1* affects chloroplast function and seedling vigor (Echt et al., 1987).

A collocation of QTL for anthesis (lines-DS), GLAD (hybrids-DS), grain yield (hybrids-WW), PHT (hybrids-WW), and NDVI (hybrids-WW) was detected in bin 1.03, indicative of the importance of this bin for the genetic control of early and general vigor (i.e. NDVI and PHT) and grain yield. Candidate genes for the response related to this chromosome region are a set of genes related to chlorophyll fluorescence (*hcf3*,*hcf31, hcf6*) and leaf color (pg^*^-N484A, pg^*^-N484B, and pg^*^-N526C), which may relate to seedling vigor.

A collocation of QTL for PHT (hybrids-WW), leaf rolling (hybrids-DS) and senescence (hybrids-DS) detected in bin 7.04 may suggests that general vigor confers stress avoidance later in the season, since the QTL for PHT was in repulsion with the QTL for leaf rolling and senescence. While PHT reflects general shoot vigor, it may also indicate root vigor (Richner et al., 1996; Hammer et al., 2009; Grieder et al., 2013) as a result of allometric root-shoot relations. Shoot vigor indicative of a vigorous root system, would indirectly allow for greater water and nutrient uptake from deeper soil layers resulting in lower stress levels and reduced leaf rolling and senescence under drought stress. This hypothesis is supported by QTL previously detected for PHT (Sibov et al., 2003) and root architecture in this bin (Tuberosa et al., 2003; Trachsel et al., 2009; Cai et al., 2012). Potential candidate genes underlying the observed response are *hcf101, hcf103*, and *hcf104*, which are related to chlorophyll fluorescence conveying sufficient assimilates and plant vigor.

### Detection of QTL constitutive across environments or consistent across populations

Although a total of 48 QTL were detected for grain yield and secondary traits, none of them was consistently detected in hybrids and lines as a result of the low correlation found among lines and hybrids and across treatments as a result of genotype-by-environment interaction, epistasis and heterosis (Mei et al., 2005; Mihaljevic et al., 2005; Szalma et al., 2007; Hallauer et al., 2010). These results highlight the need to use the testcross' phenotype in mapping studies rather than lines' as done previously (Trachsel et al., 2009, 2010), when aiming to identify QTL to be deployed in hybrids. Only one QTL detected for grain yield in lines was constitutive across treatments. Since there was low correlation of grain yield among lines and hybrids, its usefulness in breeding programs is limited. No QTL for any other trait was detected across treatments, as observed previously (Edmeades et al., 1999). Nevertheless high correlations were found across treatments for anthesis (hybrids and lines) and for PHT (lines).

One constitutive QTL was identified when QTL reported here were compared to results from another study evaluating the same population under nitrogen deficient conditions (DHpop1; Liu personal communication) and an advanced backcross population with a common parent (LPSC7F64; Trachsel et al., 2016) under DS and WW. A senescence QTL under drought (Trachsel et al., 2016) and nitrogen deficient conditions (Liu personal communication) was also detected in bin 4.05; only for lines-DS here, for two populations of hybrids under low N stress (Liu personal communication). These findings are in agreement with two QTL related to senescence detected in this bin by Belícuas et al. (2014) under rain-fed conditions. This QTL has great value for breeding as it could bring yield advantages under two common stresses occurring in tropics (i.e., drought and low soil nitrogen) through improved stay-green. Two candidate genes related to senescence have been reported in this bin. One is *SWEET15a*, which regulates sucrose translocation in the plant (Chen, 2014). The second is *nnr1*, which regulates nitrate reductase, a crucial enzyme in nitrite assimilation in plants (Rockel et al., 2002).

### Correlation between genomic prediction accuracy and trait heritability

In this study, the results showed that the prediction accuracy of grain yield under WW conditions was better than that under DS conditions. The prediction accuracy of the secondary traits were generally higher than the prediction accuracy of GY under almost all the conditions. However, a positive correlation was only observed between the genomic prediction accuracy and trait heritability for hybrids WW. Low correlation between genomic prediction accuracy and trait heritability was observed for all other treatment-by-population combinations. Since the training population was of the same size for all traits the lower prediction accuracy irrespective of the heritability could potentially be attributed to reduced phenotypic variation or large genotype-by-environment interaction (Zhang et al., 2017). Since Combs and Bernardo (2013) additionally show that prediction accuracy may also be dependent on the genetic architecture of a specific trait it is conceivable that genetic and physiological mechanisms acting under stressed conditions are responsible for the observed lack of correlation between trait heritability and prediction accuracy.

### R^2^ of QTL and prediction accuracy of GS models for grain yield and secondary traits

Prediction accuracy of GS-MAS was higher than the overall variance explained by all QTL for a trait (R^2^) in QTL-MAS for grain yield as observed previously (Meuwissen et al., 2001; Bernardo and Yu, 2007; Lorenzana and Bernardo, 2009; Mayor and Bernardo, 2009; Heffner et al., 2010; Guo et al., 2012; Zhao et al., 2012). A similar pattern was observed for secondary traits (i.e., anthesis, PHT, ASI, NDVI, senescence, leaf rolling and GLAD). From a practical point of view, strong QTL remain important in QTL-MAS, as suggested by Heffner et al. (2010). While GS-MAS requires several hundred markers, only flanking markers of target QTL are needed in QTL-MAS. Detected QTL with beneficial effects on early vigor, drought escape, grain yield and stay-green, such as the ones detected in bins 1.02, 1.03, 7.04, and 4.05 could be used in forward breeding to enrich alleles for these traits in a breeding program or for line conversions, while GS-MAS could be used in more mature breeding programs to additionally capture alleles with smaller additive effects (Heffner et al., 2010; Cao et al., 2017). Ideally selection could be carried out for major and minor additive effects simultaneously by using major QTLs as fixed factors in GS-MAS as described by Bernardo (2014).

### Best performing hybrids

To be commercially successful, a hybrid needs to perform well under non-stressed and stressed conditions. The fact that no hybrid reached the top ten under both WW and DS conditions is indicative of the difficulty to achieve high grain yield across environmental conditions due to potential physiological tradeoffs between optimal and stressed conditions. However, hybrids (LPSC7F64/CML495)DH220/CML494, (LPSC7F64/CML495)DH290/CML494 and (LPSC7F64/CML495)DH30/CML494 performed well under WW conditions (all of them in the top ten) and drought stressed conditions (all of them were within the best 22 out of 190). Their superior yield potential and stability was reflected by 12% higher grain yield relative to the commercial check (DK357) under both WW and DS conditions. Also, the hybrid (LPSC7F64/CML495)DH109/CML494, ranking 14th and 27th out of 190 under WW and DS conditions, respectively (data not shown) ranked fourth in a study where the same set of hybrids was grown under low nitrogen (Liu et al., personal communication). After further evaluations across sites, in combination with multiple testers, lines DH220, DH290 and DH30 could be released as CIMMYT maize lines for deployment in drought prone environments, while line DH190 could potentially be used in environments prone to drought and low nitrogen.

## Author contributions

ST and RB designed and conceived the experiment; CM, ES, and ST carried out the experiments; DC, YY, SC, XZ, and ST analyzed the data; DC and ST wrote the manuscript.

### Conflict of interest statement

The authors declare that the research was conducted in the absence of any commercial or financial relationships that could be construed as a potential conflict of interest.
